# Antioxidants as Protection against Reactive Oxidative Stress in Inflammatory Bowel Disease

**DOI:** 10.3390/metabo13040573

**Published:** 2023-04-18

**Authors:** Sara Jarmakiewicz-Czaja, Katarzyna Ferenc, Rafał Filip

**Affiliations:** 1 Institute of Health Sciences, Medical College of Rzeszow University, 35-959 Rzeszow, Poland; 2 Institute of Medicine, Medical College of Rzeszow University, 35-959 Rzeszow, Poland; 3 Department of Gastroenterology with IBD Unit, Clinical Hospital No. 2, 35-301 Rzeszow, Poland

**Keywords:** antioxidants, Crohn’s disease, inflammatory bowel disease, nutrients, oxidative stress, ulcerative colitis

## Abstract

Inflammatory bowel disease (IBD) belongs to a group of chronic diseases characterised by periods of exacerbation and remission. Despite many studies and observations, its aetiopathogenesis is still not fully understood. The interactions of genetic, immunological, microbiological, and environmental factors can induce disease development and progression, but there is still a lack of information on these mechanisms. One of the components that can increase the risk of occurrence of IBD, as well as disease progression, is oxidative stress. Oxidative stress occurs when there is an imbalance between reactive oxygen species (ROS) and antioxidants. The endogenous and exogenous components that make up the body’s antioxidant defence can significantly affect IBD prophylaxis and reduce the risk of exacerbation by neutralising and removing ROS, as well as influencing the inflammatory state.

## 1. Introduction

Inflammatory bowel disease (IBD) is a group of chronic diseases that follow periods of exacerbation and remission [1]. In Crohn’s disease (CD), inflammation can affect any section of the gastrointestinal tract, from the mouth through the oesophagus, stomach, small intestine, large intestine, to the rectum. Lesions are separated by healthy fragments. Inflammation is most often located in the terminal segment of the small intestine and the initial colon. In ulceratis colitis (UC), inflammation can be localised in the rectum or rectum and colon. Inflammation forms in the mucosa and submucosa, leading to ulceration. The characteristic distinguishing UC from CD is the presence of the continuation of inflammatory lesions [2]. An increase in the incidence of IBD has been observed year after year. Researchers predict that its prevalence will increase significantly in the coming years. The incidence of UC is higher in comparison to CD. The most common diagnosis of UC occurs in the third and fourth decades of life, while CD is diagnosed primarily in people in their second and third decades. Up to 16% of cases of IBD are diagnosed after age 65, while about 4% are diagnosed before age 5. They are most often diagnosed in the Caucasian race. Ashkenazi Jews of Central and Eastern European descent are a particularly vulnerable group, with a four-time higher incidence of CD than others in the Caucasian population [3,4]. IBDs are diagnosed in patients with a genetic predisposition, and there is often a correlation with specific environmental factors [1]. In addition, among the most important factors predisposing to IBD are ROS, which, as a result of the formation of oxidative stress, can lead to dysfunction of the intestinal barrier, thus increasing the activity of the immune system [5]. The main objective of this review is to collect scientific reports on the action of antioxidants in protecting against oxidative stress in IBD and to identify knowledge gaps that can inform further research.

## 2. Oxidative Stress and Reactive Oxygen Species

Oxidative stress (OS) is defined as an imbalance between the induction of reactive oxygen species (ROS) and the antioxidant components of the body’s defence system [6]. ROS are molecules composed of at least one oxygen atom and containing at least one unpaired electron. From a biochemical perspective, ROS are highly reactive compounds that interact reactively with cell organelles [7]. ROS include hydrogen peroxide (H_2_O_2_), superoxide radicals (O_2_•−), hydroxyl radicals (•OH), and singlet oxygen (O_2_). They are mainly produced as by-products of oxygen metabolism processes, but there are external triggers that contribute to their increase [8]. These triggers include exposure to the external environment (ultraviolet radiation, chemicals such as benzene and phenols), diseases (cancer, infections), and certain drugs (cyclosporine, gentamicin) [9]. Mitochondria, as the central location for oxidative phosphorylation, produce free oxygen radicals by reducing oxygen to water. Oxidative phosphorylation is considered the most efficient human process of energy production [10]. Other sources of ROS production include xanthine and flavin oxidases, as well as cytochrome P450 [11]. Enzymes such as peroxidases, xanthine oxidase (XO), NADPH oxidase (NOX), lipoxygenases (LOX), myeloperoxidase (MPO), nitric oxide synthase (NOS), and cyclooxygenases (COX) are involved in the endogenous generation of ROS by catalysing chemical reactions [12]. Among this group, NOX and XO may be involved in the pathogenesis of IBD [13]. In addition to ROS, other molecules, such as reactive nitrogen species (RNS) and reactive sulphur species (RSS), also participate in oxidative stress. RNS comes from the reduction or oxidation of nitrogen compounds, while RSS comes from the conversion of redox compounds containing sulphur. To understand the interaction and mutual interaction of ROS, RNS, and RSS, the concept of “reactive species interactome” was created [14]. The physiological level of ROS does not disturb redox balance and is beneficial for maintaining normal body homeostasis. These compounds participate, among other things, in inducing the immune system and also participate in controlling insulin secretion [15]. They mainly perform the function of signal transduction, thus stimulating the activity of cells and providing them with protection [16]. Other studies have shown that ROS are responsible for a wide range of physiological processes. These include, among others, cell differentiation and proliferation, posttranslational modification of proteins, gene expression, or adaptation to hypoxia [17]. Apoptotic pathways for programmed cell death are also functions of reactive oxygen compounds [18]. The functions described above indicate the significant role of ROS in the context of normal body homeostasis by maintaining their optimal levels in the cellular environment [19]. ROS production is a natural cellular process. To maintain proper balance, ROS levels must be balanced with antioxidants [20]. The lack of this balance due to increased ROS production and/or reduced antioxidant defence leads to oxidative stress. Under pathological conditions, it causes structural damage to lipids, proteins, and deoxyribonucleic acid (DNA) [21]. As a result, the cell loses its ability to maintain basic functions. It experiences broad-based dysfunction, which can lead to cell death [22]. A characteristic feature of IBD is the state of the mucous membrane, with significant infiltration of inflammatory cells. This infiltration is the result of the inflammatory state during exacerbation of the disease, which causes damage to the mucous membrane. In addition, constant exposure to bacteria and fungi belonging to the gut microbiota influences the state of the intestines. In the course of IBD, the microbiota is colonised much more frequently by pathogenic microorganisms in contrast to healthy individuals [23]. Throughout this process, there is excessive activation of effector lymphocytes along with increased production of pro-inflammatory cytokines. Thus, regulatory cells cannot maintain homeostasis. The result of these disturbances is a chronic uncontrolled immune response [17,24]. Oxidative stress is the initiator of many diseases, including cardiovascular, neurodegenerative, chronic lung, and kidney diseases, as well as cancers and IBD [25,26]. Therefore, the balance between ROS generation and reduction is important and essential in order to avoid cell damage. Body homeostasis is conditioned by the balance of redox, which ultimately has a crucial impact on human health and quality of life [27].

## 3. Oxidative Stress and Inflammatory Bowel Disease (IBD)

Many researchers argue that IBD is closely related to increased ROS production. Studies in animal models with induced colitis using dextran sulphate sodium (DSS) confirm the increased generation of ROS such as superoxide, hypochlorous acid, and hydrogen peroxide. At the same time, a reduction in the level of endogenous antioxidant compounds, including glutathione and superoxide dismutase, is observed [28]. Active and chronic inflammation of the mucous membrane is directly related to the generation of ROS, which serve as important signalling molecules in the context of the immune response [29,30]. Furthermore, studies show that ROS production in the microenvironment of inflammatory changes in the mucous membrane causes secondary damage, including extensive cellular and molecular damage. As a consequence, this can lead to the maintenance and consolidation of intestinal inflammation, as well as induce further cell damage [31]. Such damage increases the risk from pathogens (including through increased permeability of the intestinal barrier), which in turn may induce a renewed immune response that can initiate the development of IBD. Additionally, ROS overproduction alters intestinal absorption and disrupts intestinal peristalsis. Therefore, in recent years, the number of studies on oxidative stress as a major intermediate factor in the development of IBD has increased [32]. The protein complex acting as a transport factor known as the NF-kB (nuclear factor kappa-light-chain-enhancer of activated B cells) is an important regulator in many diseases. Studies have shown that it is improperly activated in patients with IBD [33]. A study in mouse models has shown that an antioxidant drug that inhibits NF-kB activity can alleviate symptoms of colitis [34]. Recently, it has been observed that environmental factors such as high consumption of saturated fats and refined sugars, excessive use of antibiotics, or even stress resulting from daily life contribute to a high risk of IBD [35]. Cigarette smoke has been reported to greatly reduce endogenous antioxidant activity in the colon [36]. In IBD, oxidative stress is not limited to the digestive tract, but also has systemic effects in the form of extraintestinal manifestations [37,38]. Additionally, oxidative stress may be involved in the carcinogenesis process in patients with IBD [39]. A study reports that *Helicobacter pylori* may influence neutrophil induction to generate ROS, which ultimately contributes to the onset of gastric cancer [40].

## 4. Antioxidants

**Figure 1:**
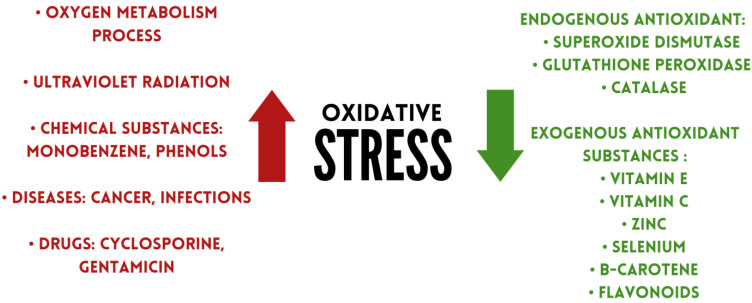
Factors affecting oxidative stress levels.

Antioxidants are substances that can remove damage caused by oxidative stress, or prevent or delay it [41]. Under physiological conditions, antioxidants regulate the production of free radicals [42]. Based on their occurrence, antioxidants can be divided into two groups: endo- and exogenic. The first group includes superoxide dismutase, glutathione peroxidase, catalase. Exogenous antioxidants include flavonoids, vitamins, and minerals, among others (Figure 1) [43]. 

### 4.1. Exogenous Antioxidant Substances

#### 4.1.1. Vitamin E

Vitamin E comprises four tocopherols (α, β, γ, δ) and four tocotrienols (α, β, γ, δ). The antioxidant properties of α-tocopherol are comparable to α-tocotrienol [44]. A-tocopherol is the best bioavailable form of vitamin E due to α-tocopherol transfer protein (α-TTP), which has a 100% affinity for α-tocopherol and is the main determinant of α-tocopherol concentration in plasma [45]. Vitamin E can modulate prostaglandin E2 (PGE2) production [46]. PGE2 is involved in enhancing cytokine signalling through gene regulation. It also facilitates Th1 differentiation and Th17 expansion [47]. Additionally, it promotes the production of IL-22 by Th22 cells. In contrast, the improvement of PGE2 inflammation occurs mainly through EP2 and EP4 receptors [48]. In a study by Liu et al., researchers examined the effects of α-tocopherol (αT) and γ-tocopherol (γT) on colonic inflammation and intestinal barrier function. They conducted the study in animal models in which inflammation was induced by the administration of DSS (dextran sulphate sodium). They observed that both compounds exhibited anti-inflammatory effects, but had different effects on intestinal microflora. The researchers found that under pathological conditions γ-tocopherol-rich tocopherols (γTmT) change intestinal microflora to a more favourable composition, while they have no effect on microorganisms in healthy individuals. Both α- and γ-tocopherol have beneficial effects on improving intestinal barrier function [49]. In another study, Lee observed that vitamin E, when given together with pentoxifylline (PTX), could potentiate the effects of the drug. PTX and vitamin E have been shown to reduce the induction of fibrinogen marker expression, suggesting that vitamin E should be considered for inclusion in antifibrotic intestinal therapy in patients with IBD [50]. Chen et al. in their study examined single nucleotide polymorphisms and observed that genetically higher levels of vitamin E were associated with a reduced risk of UC [51]. Other researchers, through RNA sequencing, observed that AHRR (AHR repressor) deficiency reduced IEL (intestinal intraepithelial lymphocytes) representation. In addition, they found the occurrence of oxidative stress in Ahrr -/- IELs. Supplementing with vitamin E and selenium restored redox balance [52]. Vitamin E, together with other compounds with antioxidant activities, may be a potential protective factor against colorectal cancer [53]. Fan et al. compared the effects of vitamin E and D on UC in rats induced by DSS. Both vitamins showed anti-inflammatory effects. Vitamin E, when administered at 30 IU/kg, reduced the levels of inflammatory mediators, i.e., IL-6, IL-12, IL-18, TNF-α (tumor necrosis factor α). The authors indicate that studies of the effect of vitamin E in humans are necessary [54]. Newly developed vitamin E derivatives can also induce suppression of keratinocyte-derived chemokine and IL-6, which could be used to treat UC [55]. The main sources of vitamin E in food are vegetable oils, nuts, and seeds [56].

#### 4.1.2. Vitamin C

L-ascorbic acid is a compound that is not synthesised in the human body, so it must be supplied from exogenous sources. Vitamin C exhibits immunomodulatory and protective effects against ROS [57]. It can have a protective effect on the endothelium, among other things, by decreasing ROS in endothelial cells or neutralising the nitrate tolerance phenomenon [58]. It can act as a cofactor for mono and dioxygenase enzymes [59,60]. The hydroxyl groups of ascorbate in the lactate ring are electron donors and proton donors; they convert to the diketone moiety of dehydroascorbate (DHA), and therefore have a protective effect on cells. Such action of hydroxy groups is shown against superoxide radicals, singlet oxygen, and hydrogen peroxide [61]. Due to molecular stabilisation, the resulting ascorbyl radical is hardly reactive [62]. A high concentration of vitamin C has a protective effect on neutrophils against ROS. It also affects neutrophil leukocyte chemokinesis and chemotaxis [63]. In IBD, up to one fifth of patients with active inflammation may be vitamin C deficient, which may also be associated with feelings of increased fatigue and impaired wound healing [64]. The cause of vitamin C deficiency in patients may be due not only to the active form of the disease, but also to the avoidance of fruit and vegetable consumption [65]. A study by Miyake et al. showed that a higher intake of vitamin C and vegetables may be associated with a lower risk of UC [66]. Other researchers have reached similar conclusions [67]. Patients with CD also show reduced vitamin C intake [68]. Jo et al. studied the effect of vitamin C deficiency in induced inflammatory bowel disease by administering DSS to mice. They observed that deficiency of the compound caused a decrease in mucin, while it increased IL-6 production and oxidative stress [69]. The *SLC23A1* polymorphism may result in a decreased activity of the ascorbate transporter and its reduced intracellular amount [70]. Good dietary sources of vitamin C include berries, citrus fruits, parsley [71].

#### 4.1.3. Zinc

Zinc is a trace element that must be supplied to the body in order for it to function properly. Zinc deficiencies can lead to malfunctioning of T and B lymphocytes and to abnormal maturation and differentiation of them [72]. In addition, they can cause decreased phagocytosis and PMN (polymorphonuclear cells) chemotaxis, and also affect monocyte adhesion to the endothelium [73]. Zinc has also been shown to preserve redox metabolism. An example is the increase in intracellular zinc in granulocytes caused by H_2_O_2_. Zinc can also be released in increased amounts from metallothionein (MT) through ROS induction [74]. Zinc can increase IFN-γ (interferon gamma) secretion from peripheral blood mononuclear cells (PBMCs) [75]. Deficiency of the element can lead to increased production of TNFα and IL-6 [76]. The antioxidant activity of zinc occurs indirectly. Its antioxidant functions are multiple, including increasing glutathione (GSH) production or as a cofactor of antioxidant enzymes [77]. The element is also essential for maintaining normal intestinal barrier function, as its deficiency can reduce the function of the tight junction resulting in increased permeability. Additionally, the repair of the intestinal barrier requires the presence of zinc [78,79]. Zinc is also responsible for the proper functioning of intestinal alkaline phosphatase [80]. Deficiencies are more common in patients with IBD than in the general population [81]. In patients with IBD, microelement deficiencies can increase the risk of complications of the disease, as well as hospitalisation [82]. Therefore, screening is recommended, especially during disease exacerbations, to identify possible deficiencies [83].

#### 4.1.4. Selenium

Selenium was discovered in 1817 [84]. Biologically, it is found in the form of 25 selenoproteins and occurs in humans as an element with immunomodulatory effects, among others. It mainly neutralises organic hydroperoxides and hydrogen oxides [85]. It has been shown to act on immune cells, such as NK cells and T lymphocytes, by affecting selected cell signalling pathways or antioxidant functions [86,87]. The element also modulates redox signalling and counteracts ROS [88]. The main compound in the selenoprotein group is glutathione peroxidase (GSH-Px). It consists of 4 units containing selenocysteine, which are antioxidant [89]. It can regulate free radical production when there is inflammation [90]. In addition, it can support immunoglobulin production [91]. Selenium is also essential for the metabolism of some intestinal microorganisms [92]. Cytoplasmic ROS activate the NF-κB signalling pathway and are subsequently involved in the expression of IL-2 and IFN-γ. Therefore, it is important to monitor selenium levels in IBD [93,94,95]. Yan et al. tested whether there was a correlation between serum selenium levels and disease activity in CD patients. After including 135 patients in the study, they observed that serum concentrations of the element were inversely correlated with the severity of the disease course, indicating that selenium could be a factor along with other factors for monitoring disease activity [96]. Some researchers indicate that it is possible to enhance the effect of a probiotic by adding selenium to it, which may also mitigate the inflammation that occurs [97,98,99]. This could be due, among other things, to an increase in *SIRT1* gene expression [100]. Keshteli et al. in their study observed that a diet containing anti-inflammatory ingredients altered the composition of the intestinal microflora in patients with UC and led to metabolic changes, which consequently supported the maintenance of clinical remission [101]. In addition, adequate selenium levels can reduce the risk of cardiovascular disease in patients with IBD [102]. Short et al. in their study observed that selenoprotein P (SEPP1) has a significant role in the regulation of intestinal homeostasis and thus the occurrence of inflammation and indirectly colorectal cancer [103,104].

#### 4.1.5. Betacarotene

Β-carotene is a vitamin A provitamin and belongs to the carotenoid group. It has a C40 in its structure including two β-ion rings [105]. By scavenging superoxide radicals and quenching singlet oxygen, it is considered a compound with antioxidant properties [106]. The antioxidant properties of the compound depend on its conformation. Hydrogen abstraction reactions are more exothermic in water compared to gaseous media [107]. Β-carotene shows positive effects in many diseases, such as diabetes and skin diseases [108,109]. Carotenoids also show beneficial effects on the gastrointestinal tract [110]. Honarbakhsh et al. investigated whether carotenoids can have a positive effect on improving intestinal dysfunction. They showed that in the presence of vitamin A deficiency, the administration of β-carotene can reduce intestinal ROS and levels of pro-inflammatory cytokines. In addition, the compound may also have the effect of reducing the permeability of the intestinal barrier [111]. Cheng et al., using epithelial cells in vitro, also observed an improvement in intestinal barrier function by enhancing tight junction function. They also found that with LPS- (lipopolysaccharide) induced colitis, β-carotene can reduce inflammation by down-regulating the toll-like receptor 4 (TLR4) pathway [112]. In addition, provitamin A can exhibit IL-6 and TNF-α lowering abilities [113]. Inflammatory bowel disease can also be alleviated by decreasing PGE2, nitric oxide (NO) production, and modulation of certain signaling pathways [114,115]. Other studies, in animal models, have shown that β-carotene administration can modulate the composition of the intestinal microbiota, which could significantly benefit patients with IBD [116,117]. Good dietary sources of β-carotene include vegetables (carrots, kale, parsley, chard) and fruits (apricots, melon) [71].

#### 4.1.6. Flavonoids

Flavonoids are compounds made up of a benzopyrone ring that contains polyphenolic or phenolic groups. They have a variety of uses and actions [118]. The main groups of substances belonging to the category of flavonoids are: anticyanins (examples of bioactive substances: cyanidins, pelargonidins), flavanols (e.g., catechin, epicatechin), flavonols (e.g., quercetin, kaempferol), flavones (e.g., luteolin, apigenin), flavanones (e.g., naringenis, naringin), and isoflavones (e.g., daidzein, genistein) [119,120]. Due to the presence of a hydroxyl group in the β-ring and a double bond, flavonoids exhibit antioxidant abilities against peroxynitrite, superoxide, or hydroxyl radicals [121]. The antioxidant role of flavonoids is exerted by chelating metal ions, trapping reactive oxygen species, detoxifying enzymes, and increasing the production of antioxidant enzymes [122]. They also inhibit the expression of pro-inflammatory mediators such as the NF-κB cascade, and inhibit the release of pro-inflammatory cytokines [123]. In addition to their pro-inflammatory properties, the compounds show the ability to regulate tumour-associated macrophages (TAMs) [124]. The anti-inflammatory effects of flavonoids focus primarily on inhibiting the activation of intracellular protein complexes containing PRRs (pattern recognition receptors) and inflammatory molecules. This occurs by decreasing the expression of components of the inflammasome, resulting in inhibition of caspase-1 activation and the secretion of pro-inflammatory cytokines [125]. Flavonoids also show non-direct effects on the gut. In their work, Wang et al. show that citrus flavonoids can exert positive effects on maintaining normal intestinal barrier functions by regulating the expression of TJ (tight junction) expression. They mainly point to nobiletin as the bioactive component of flavonoids, which shows effects similar to those of an anti-inflammatory drug. In addition, citrus flavonoids show regulatory effects on mucin expression and secretion and on shaping the composition of intestinal microflora [126,127]. Due to their properties, flavonoids may exert beneficial effects on the course of IBD by, among other things, protecting against functional and morphological changes in the vascular endothelium [128]. Furthermore, they may counteract colonic inflammation by activating the AhR/Nrf2/NQO1 pathway as well as limiting the action of the NLRP3 (NLR family pyrin domain-containing-3) inflammasome [129]. Due to all these factors, antioxidants can reduce the disease activity index [130,131]. The main sources of flavonoids in food are herbs, vegetables, fruits, nuts, cereals, coffee, and tea [132].

### 4.2. Endogenous Antioxidant Substances

Despite the fact that excessive and uncontrolled oxidative stress has destructive properties for the digestive system, antioxidant defence systems can counteract the undesirable effects of ROS [133,134]. The main defence mechanism of the body involves the production of endogenous antioxidants, including superoxide dismutase (SOD), glutathione peroxidase (GPX), and catalase (CAT) [135].

#### 4.2.1. Superoxide Dismutase

Superoxide dismutase (SOD) is responsible for transforming superoxide radicals into hydrogen peroxide (H_2_O_2_) and molecular oxygen (O2) [136]. Hydrogen peroxide is subsequently converted by catalase and glutathione peroxidases [137]. The excessive and uncontrolled production of H_2_O_2_ can be potentially harmful to cells. In contrast, an optimal concentration of hydrogen peroxide may have a signalling effect [138]. Superoxide dismutase exists in three isoforms: SOD1, SOD2 and SOD3. SOD1 is present mainly in the cytosol of liver and kidney cells, as well as in the central nervous system and erythrocytes. SOD2 is predominantly found in mitochondria. SOD3 is found in blood serum, tissues, and body fluids (including synovial fluid and cerebrospinal fluid) [139]. A study showed that SOD activity was elevated in rats with acetic acid-induced UC compared to the control group. It appears to be a defensive reaction against oxidative damage under inflammatory conditions caused by the disease [140]. A study found that patients with inactive CD have a higher activity of SOD compared to those with active CD. This suggests that endogenous antioxidant defence during disease exacerbation is impaired. Furthermore, in the same study, Szczeklik et al. demonstrated that the level of C-reactive protein (CRP) in the CD group was inversely correlated with SOD activity in serum [141]. Another study indicated that the SOD concentration was statistically significantly lower in 42 patients with CD compared to healthy individuals. A limitation of this study was the lack of division of CD patients into a group with exacerbation of the disease and a group in remission. The authors of the study concluded that CD patients are more susceptible to oxidative stress than healthy individuals [142]. Mohammadi et al. also found a decrease in SOD activity in CD patients, as well as UC patients [143]. Zielinska et al. showed a significant decrease in SOD activity in patients with IBD compared to the control group, while also noting a significant decrease in glutathione peroxidase (GPX) only in patients with CD [144]. A study involving 40 mice with IBD showed that supplementation with a strain of *Lactobacillus* with activity similar to SOD was much more effective in alleviating inflammation compared to strains with activity similar to catalase [145]. A similar study, this time using the strain *Bifidobacterium bifidum* BGN4-SK, which was created to produce SOD and CAT, was conducted in mice with DSS-induced intestinal inflammation. *B. bifidum* BGN4-SK was found to effectively increase antioxidant potential, inhibit colon inflammation, and protect the integrity of the large intestine epithelium [146]. In recent years, a great deal of attention has been paid to exogenous supplementation of antioxidants as a therapeutic approach in IBD to reduce ROS, indicating that antioxidants have enormous potential in both preventing and supporting the treatment of inflammatory diseases. Liang et al. conducted a study on mouse models with DSS-induced colitis, which received oral capsules containing SOD and CAT. The results showed that supplementation with antioxidant capsules can effectively reduce ROS and also inhibit the secretion of pro-inflammatory cytokines, ultimately reducing inflammation in the colon [147].

#### 4.2.2. Glutathione Peroxidase (GPX)

Glutathione peroxidase is a broad family of compounds with peroxidase activity [148]. GPX has the ability to catalyse the conversion of glutathione to oxidised glutathione (GSH) and can also reduce H_2_O_2_ to water molecules and lipid hydroperoxides to stable alcohols. Humans have eight GPX isoforms, many of which contain selenocysteine residues [149]. GSH, as a soluble antioxidant, has been shown to be less active in experimental mouse models of inflammatory bowel disease [150]. A study in mouse models of Crohn’s disease and ulcerative colitis reported that GPX2 plays a significant role in antioxidant defence against oxidative stress and inflammation in the intestinal mucosa, but is also significantly induced in stomach cancer [151]. Studies show that concurrent mutations in GPX1 and GPX2 in mice produce symptoms similar to those of patients with IBD, suggesting that this is due to oxidative damage in the digestive tract [152]. The study by Rana et al. showed a significant decrease in reduced glutathione (GSH) activity in erythrocytes of 81 patients with UC compared to a healthy control group. Additionally, they found higher levels of lipid peroxidation in patients, which may be a consequence of oxidative stress [153]. Krzystek-Korpacka et al. examined 174 patients diagnosed with IBD and found a statistically significant decrease in GPX in patients with active Crohn’s disease and ulcerative colitis compared to healthy individuals and those in remission [154].

#### 4.2.3. Catalase

Catalase is located mainly in peroxisomes [155]. CAT is responsible for breaking down H_2_O_2_ into water and molecular oxygen, thus preventing cell damage resulting from the Fenton reaction. In the Fenton reaction, which requires the presence of transition metal ions such as iron or copper, a highly reactive hydroxyl radical (HO) may be formed. In some cases, where catalase is absent, its functions can be performed by glutathione peroxidase [156]. Catalase can also act in a so-called peroxidative mode, in which its functions involve the breakdown of small substrates such as methanol or formate [157]. Another important function of catalase is apoptosis [158]. A study showed that CAT activity in erythrocytes increases in patients with UC [153]. In contrast, another analysis found persistent inhibition of CAT activity in mononuclear cells in patients with CD [159]. On the basis of this, Iborra et al. showed that the constant decrease in CAT observed in CD patients may be due to genetic changes. Various genetic mechanisms that inhibit this antioxidant may contribute to the pathophysiology of CD [160].

Table 1 summarizes the mechanisms of action of endo- and exogenous antioxidants.

## 5. Limitations

There are a small number of studies in humans on the effects of specific antioxidants on periods of remission and exacerbation of IBD. In addition, the methodology of studies is often not defined in terms of homogeneity of groups, age, or drugs used. There are no conclusive studies on the role of oxidative stress in the pathophysiology or progression of IBD. It should be noted that oxidative stress may result not only from IBD itself, but also from other factors, such as diet (low fruit and vegetable intake), low physical activity, malnutrition, or psychological stress. Any of these factors can interfere with the interpretation of scientific findings.

## 6. Conclusions

Due to the possibility of nutritional deficiencies in patients with IBD resulting from poor absorption, chronic inflammation, and/or reduced consumption of certain foods, nutritional therapy should be an integral part of treatment [163,164,165]. Many studies suggest paying particular attention to compounds with antioxidant properties, such as vitamins E and C, zinc, selenium, carotenoids, flavonoids, and many others. It seems that factors such as genetic, immunological, microbiological, and environmental factors, along with oxidative stress, play a significant role in the initiation and development of IBD. This is due to the disturbance and loss of homeostasis between the gut microbiota and the immune system of the patients. Oxidative stress is a physiological process in which cells experience an imbalance between the generation of reactive oxygen species and the body’s ability to neutralise and remove them. Due to the wide spectrum of oxidative stress in IBD, many attempts have been made in recent years to analyse individual antioxidants to find an alternative treatment or support therapy method for patients with UC and CD. The results obtained so far suggest potential benefits from their actions; however, more research is necessary to clarify the mechanisms connecting oxidative stress with the onset of IBD.

## Figures and Tables

**Table 1:** Summarising the mechanism of action of antioxidants.

	Substance	Mechanism of Action
Endogenous antioxidants	Superoxide dismutase	- conversion of superoxide radical to hydrogen peroxide (H_2_O_2_) and molecular oxygen (O_2_) [136] - lowering C-reactive protein levels [141] - alleviating symptoms associated with colitis by inhibiting production of pro-inflammatory cytokines [147] - SOD3 (Superoxide dismutase 3) -> regulation of T lymphocyte differentiation [161]
	Glutathione peroxidase (GPX)	- catalysis of glutathione to oxidised glutathione [149] - reduction of H_2_O_2_ to a water molecule [149] - reduction of lipid hydroperoxides to stable alcohols [149] - regulation of Th17 lymphocyte differentiation [162] - inducing the production of regulatory lymphocytes [162]
	Catalase	- decomposition of H_2_O_2_ to water and molecular oxygen [156] - degradation of small substrates (methanol or formate) [157] - insufficient levels of catalase can lead to suppression of autophagy-dependent cell death [160] - the persistent reduction in the catalase enzyme observed in CD patients may be due to genetic alterations [160]
Exogenous antioxidants	Vitamin E	- modulation of PGE2 production [46] - reduction of IL-12, IL-18, TNF-α, IL-6 with vitamin E supplementation [54,55] - vitamin E supplementation reduced the decrease in ZO-1, thereby affecting the deterioration of intestinal barrier function [49]
	Vitamin C	- effects on chemokinesis and chemotaxis of neutrophil leukocytes [63] - Vitamin C deficiency can increase IL-6 production [69]
	Zinc	- deficiency can lead to decreased phagocytosis, PMN (polymorphonuclear cells) chemotaxis [73] - deficiency can lead to increased (TNF)-α and IL-6 [76]
	Selenium	- Mainly neutralises organic hydroperoxides and hydrogen oxides [85]. - Acts on immune cells, e.g., NK cells, T lymphocytes, by affecting selected cell signalling pathways or antioxidant functions [86,87]
	Betacarotene	- scavenges superoxide radicals and quenches singlet oxygen [106] - can reduce inflammation by downregulating the toll-like receptor 4 (TLR4) pathway [112] - IL-6 and TNF-α lowering abilities [113] - reduction of prostaglandin (PG)E2, nitric oxide (NO) production [114,115]
	Flavonoids	- inhibition of the NF-κB cascade [123] - chelation of metal ions, uptake of reactive oxygen species, production of detoxification enzymes [121]
